# e-Babylab: An open-source browser-based tool for unmoderated online developmental studies

**DOI:** 10.3758/s13428-023-02200-7

**Published:** 2023-08-24

**Authors:** Chang Huan Lo, Jonas Hermes, Natalia Kartushina, Julien Mayor, Nivedita Mani

**Affiliations:** 1 School of Psychology, University of Nottingham Malaysia, Semenyih, Malaysia; 2 Department of Developmental Psychology, Institute of Psychology, University of Göttingen, Waldweg 26, D-37073 Göttingen, Germany; 3 Center for Multilingualism in Society across the Lifespan (Multiling), University of Oslo, Oslo, Norway; 4 Department of Psychology, University of Oslo, Oslo, Norway; 5 Leibniz ScienceCampus Primate Cognition, Kellnerweg 4, D-37077 Göttingen, Germany; 6 Department of Psychology of Language, Institute of Psychology, University of Göttingen, Goßlerstr. 14, D-37073 Göttingen, Germany

**Keywords:** Browser-based testing platform, Developmental research, Online studies, Unmoderated testing

## Abstract

The COVID-19 pandemic massively changed the context and feasibility of developmental research. This new reality, as well as considerations about sample diversity and naturalistic settings for developmental research, highlights the need for solutions for online studies. In this article, we present e-Babylab, an open-source browser-based tool for unmoderated online studies targeted for young children and babies. e-Babylab offers an intuitive graphical user interface for study creation and management of studies, users, participant data, and stimulus material, with no programming skills required. Various kinds of audiovisual media can be presented as stimuli, and possible measures include webcam recordings, audio recordings, key presses, mouse-click/touch coordinates, and reaction times. An additional feature of e-Babylab is the possibility to administer short adaptive versions of MacArthur–Bates Communicative Development Inventories (Chai et al. *Journal of Speech, Language, and Hearing Research*, *63*, 3488-3500, 2020). Information pages, consent forms, and participant forms are customizable. e-Babylab has been used with a variety of measures and paradigms in over 12 studies with children aged 12 months to 8 years (*n* = 1516). We briefly summarize some results of these studies to demonstrate that data quality, participant engagement, and overall results are comparable between laboratory and online settings. Finally, we discuss helpful tips for using e-Babylab and present plans for upgrades.

The year 2020, and the COVID-19 pandemic that impacted societies across the world since, has changed the face of academia, especially in terms of increased focus on digital online formats for both teaching and research. The shutdown of laboratory testing facilities across the globe had many researchers scrambling for online tools to continue data collection in the midst of a pandemic. While this renewed drive for conducting studies online was made further apparent with special thematic sessions on platforms enabling online studies being introduced in major conferences, such sessions also served to highlight the many initiatives already in place to allow for conducting studies online. In psychological research with adult participants, the launch of Amazon’s Mechanical Turk truly opened the door for psychological research to go online on a large scale with access to over 250,000 users from different countries, who could choose to participate in studies in exchange for compensation (Robinson et al., 2019). Furthermore, in recent years, a number of tools have been set up to enable online data collection for behavioral research (e.g., *gorilla.sc*, Anwyl-Irvine et al., 2020 and *jsPsych.org,* de Leeuw, 2015) and for developmental research with children and infants (e.g., *TheChildLab.com*, Sheskin & Keil, 2018; *Lookit.mit.edu*, Scott & Schulz, 2017 and *discoveriesonline.org*, Rhodes et al., 2020) as well as platforms to encourage participation in online developmental studies (e.g., https://childrenhelpingscience.com/; https://kinderschaffenwissen.eva.mpg.de/). While issues associated with conducting studies online have been discussed (Hewson et al., 1996; Kraut et al., 2004), there are also a number of associated advantages that are likely to sustain interest in conducting studies online in post-pandemic times (see also Zaadnordijk et al., 2021).

## Benefits and risks of conducting studies online

Conducting studies online allows for greater diversity in the population sample recruited (Gosling et al., 2004). This is especially important in the wake of recent understanding of the diversity problem in traditional psychological research, where claims about the human population at large are made from a sample of western, educated, industrialized, rich, and democratic societies (the WEIRD problem, Henrich et al., 2010; Nielsen et al., 2017). This may be further amplified in developmental research, where research participation places additional demands on caregivers who are already pressed for time and may, therefore, self-select for caregivers from affluent backgrounds who have the time and resources to travel to a laboratory with their child to participate in a study for oftentimes little monetary reward. Relatedly, online data collection reduces constraints relating to the geographical location of the research institution, such as regional or even national borders (Lourenco & Tasimi, 2020; Sheskin et al., 2020), and allows for the same study to be run in different countries, thus paving the way for cross-cultural collaborations (cf. the current ManyBabies At Home initiative^1^). Where needed, study material can be translated into different languages, while retaining the same study setup, allowing for direct replications across different linguistic environments (e.g., across different countries). Furthermore, since studies are administered through digital devices, such as smartphones, tablets, and laptops, studies are considerably more experimenter-independent than laboratory studies, such that experimenter effects can be kept to a minimum. With regard to promoting diversity in research output, conducting studies online also allows for greater equality in terms of research output, allowing departments and laboratories with fewer resources access to larger sample sizes. Finally, and especially with regard to developmental research, conducting studies online allows research to take place in the child’s natural environment, bringing developmental research “into the wild.” Thus, generally, and especially in times of the COVID-19 pandemic, there is a need to develop an efficient infrastructure for conducting studies online (Sauter et al., 2020).

On the other hand, there are also issues associated with conducting studies online that bear mentioning and keeping in mind while planning online versus in-person studies. For instance, while online studies may allow for greater geographical diversity in population samples, participation is limited by issues related to access to reliable internet and appropriate devices, and technological literacy. Furthermore, data quality may also be impacted by differences across devices and software used (e.g., differences in response lag across devices). It is, therefore, important to weigh the benefits against the risks of conducting studies online for each task individually when deciding between online and in-person testing.

## Moderated versus unmoderated online studies

Online studies can either be moderated (i.e., involving live interaction with an experimenter through video chats) or unmoderated (Sheskin et al., 2020). Here, we outline the pros and cons of moderated versus unmoderated testing in developmental research involving children and infants, although we note that the need for a moderator may depend entirely on the task and paradigm under consideration.

While indispensable in certain paradigms (e.g., clinical assessments, or paradigms with adaptive procedures based on verbal answers), moderated online testing has the advantage of a more natural social interaction setting, as experimenters are able to take full control of the procedure and respond individually to each child (without requiring caregivers and children to be physically present at the laboratory). On the other hand, moderated testing paradigms necessitate scheduling of testing times and dates and cannot be conducted flexibly at the whim of the caregiver. Furthermore, such paradigms are more experimenter-dependent, raising issues of standardization.

Unmoderated online testing, on the other hand, implies that the procedure is fully automated and includes no live interaction. The fully automated procedure comes with high standardization and associated advantages (e.g., replicability, geographical flexibility). Families can also participate at their convenience without the need to arrange test dates. As multiple sessions can be run in parallel, there is no need for separate one-to-one sittings, thus freeing up resources in terms of experimenter hours. Nevertheless, unmoderated testing lacks the aspects of natural social interaction and the possibility to adapt the procedure to each child (e.g., to customize the pace of the study). In addition, unmoderated testing does not provide the experimenter with information about the context in which the study takes place and potential factors that may impair children’s responding (e.g., the presence of a young sibling in the same room who may be distracting the child participant or providing some of the responses to the task). Further, such paradigms are increasingly infiltrated by “bots” (software applications masquerading as human participants) or “farmers” (who attempt to bypass location restrictions) that seriously impact data quality (Chmielewski & Kucker, 2020). While these can potentially be addressed using video captures, this may also lead to cases of individuals who did not consent to being recorded (e.g., siblings) accidentally being captured on video, which would need to be clarified post hoc (see below how e-Babylab addresses this issue). Thus, as with deciding between online and in-person testing, researchers must weigh the risks and benefits of moderated and unmoderated testing paradigms in planning their studies.

## Key requirements for unmoderated online developmental studies

Here, we highlight four basic requirements which are, from our point of view, important for unmoderated online developmental studies. First, the tool needs to offer the possibility to record webcam captures of the test sessions to allow implicit and explicit responses (e.g., gaze direction, verbal answers, pointing gestures) to be captured and data quality to be evaluated. This is particularly important in research with young children who may not be able to provide manual responses (e.g., screen touches) or reliable spoken or written responses. Second, the tool needs to be browser-based so that parents can easily access the studies without extensive computer know-how or having to install specific software. Third, we consider it critical that the data collected from young children are hosted on the research group’s server without the involvement of commercial third-party or external services, to ensure that the data are securely stored in accordance with increasingly strict data protection requirements that have been adopted in many countries. A platform that uses no external data storage services and runs solely on servers of the local university may better assuage doubts and concerns of parents regarding data security and thus lower the thresholds for participation and increase acceptance for online studies. Finally, given that many researchers do not have the required skills to independently program online studies, the tool needs to include an intuitive graphical user interface (GUI) that allows even those without programming skills to create studies based on standard paradigms of developmental research with young children and infants.

Some of these features are incorporated to a varied extent in other tools already in existence, highlighting their importance for developmental research, or online research more generally (e.g., TheChildLab.com, Sheskin & Keil, 2018), and unmoderated online testing (e.g., *gorilla.sc*, Anwyl-Irvine et al., 2020; *jsPsych.org*, de Leeuw, 2015; *Lookit.mit.edu*, Scott & Schulz, 2017; and *discoveriesonline.org*, Rhodes et al., 2020). These tools differ with regard to their functions and capabilities, the programming skills required for study creation, costs, whether or not they are open source, and where the servers storing personal and audiovisual data of participants are located (see Appendix 1 for an overview of tools for unmoderated online testing that are actively maintained, i.e., with updates in 2020).

To our knowledge, no present tool is capable of meeting all four requirements highlighted in this section. Thus, we developed and tested e-Babylab, a new online tool which we present in this article. The tool is open source, with the source code available at https://github.com/lochhh/e-Babylab. The user manual is available at https://github.com/lochhh/e-Babylab/wiki. In the following sections, we will present key aspects of e-Babylab, the online study procedure from the participant’s point of view, and an overview of the GUI for study creation from the experimenter’s point of view. We will then describe several paradigms that have been successfully tested with the tool, followed by more details of a targeted replication of an established paradigm. Details of the technical underpinnings of the tool can be found in Appendix 2.

## e-Babylab

### Key aspects

e-Babylab is a web application that does not require the installation of any software other than a browser. The tool offers a high degree of flexibility for creating browser-based studies, including short adaptive versions of the MacArthur–Bates Communicative Development Inventories (CDI), and has been successfully applied to a wide range of paradigms. Its intuitive GUI allows users to create, host, run, and manage online studies without any programming experience. Studies are highly customizable and can be translated into any language.

A variety of audiovisual media (video, audio, and image files) can be presented as study material. Measures that can be recorded include key presses, click, or touch coordinates (and response latencies of these measures), and audio or video captures via the participant’s webcam and microphone. Video and audio captured in each trial allow for coding of verbal answers and for manual coding of gaze direction, facial expressions, and gestures. Media files are preloaded in the browser to enable better synchronization between the presented material and the media captures. Video and audio captures are transferred directly to the server set up at the local research facility via a secure connection (TLS, 256-bit). Since, as described above, unmoderated testing comes with the disadvantage of a lack of control over the procedure of each individual session, these media captures can also be used for post hoc evaluation of data quality (e.g., identification of parental intervention).

Recording of video data at families’ homes involves very sensitive data. Therefore, we implemented the following features to prevent the recording of private material not intended for upload. Throughout the study, an exit button is permanently visible at the lower right corner with which families can terminate or pause the study at any time. Furthermore, a maximum allowed duration (or timeout) can be set both at the study level (i.e., the maximum allowed duration for the entire study) and at the trial level. If a timeout is met, recording will stop and families will be redirected to either the end page or an optional pause page that allows them to either resume the study or proceed to the end page, where families have the option to approve the processing and use of their data or have all their data removed immediately.

### Study procedure

**Fig. 1:**
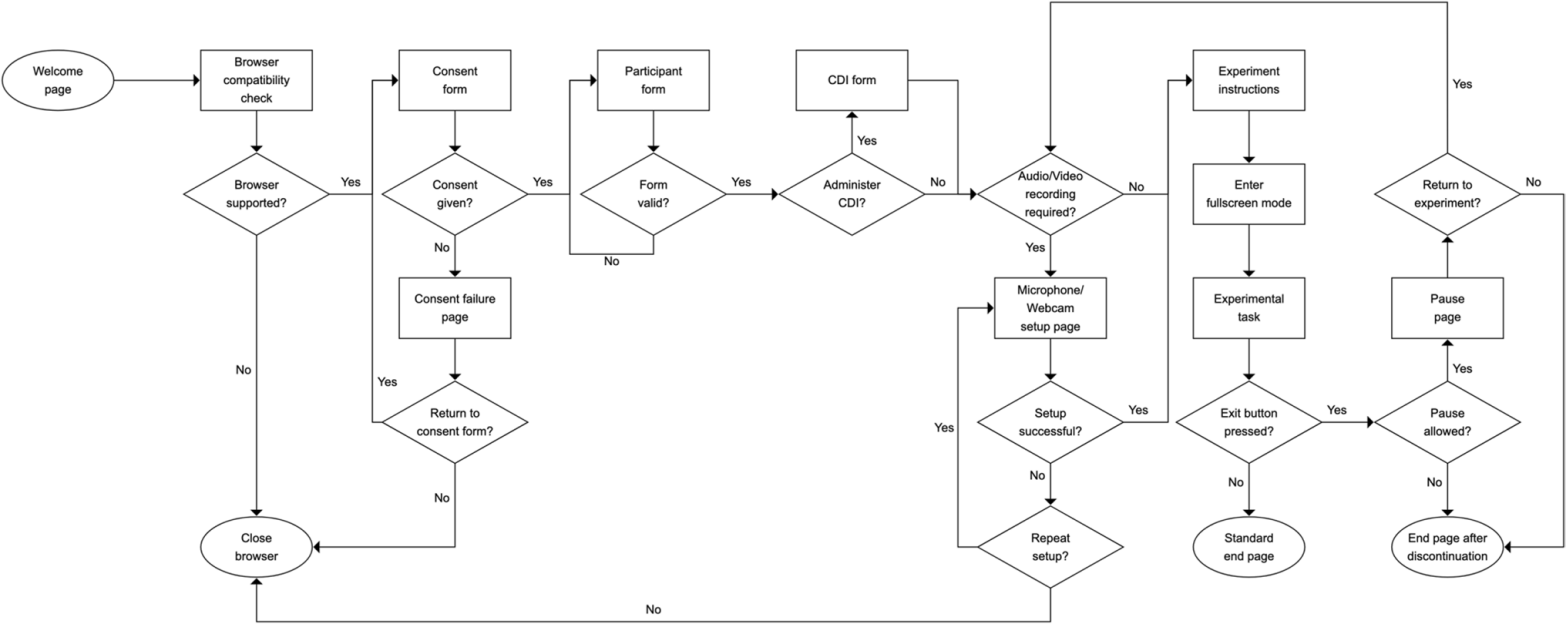
Study procedure

Figure 1 illustrates the procedure for a study created in e-Babylab. The general idea is that children will sit on their parent’s lap during the study. In the case of older children, parents may leave the study session once the pre-study steps are completed and consent is provided. Families will need a device (e.g., laptop, tablet, or smartphone) with Google Chrome or Mozilla Firefox installed and an internet connection. At present, experiments programmed with e-Babylab are only compatible with these web browsers for desktop^2^ and Android (but not iOS—see “Media recording” in Appendix 2). These browsers made up about 82% of the Android and desktop browser market share worldwide in 2020 (NetMarketShare, 2021). Depending on the study and the responses to be captured during the study, a webcam and/or a microphone may be required.

Each study is accessed via a Uniform Resource Locator (URL) and begins with a page containing general information and requirements of the study and the testing procedure. This is followed by an automatic browser compatibility check, the consent form, the participant form,^3^ and optionally the CDI form. If the study involves audio or video recording, a microphone and/or webcam setup step is included. Otherwise, the setup step is omitted. Here, the browser first requests the participant’s permission to access their microphone and/or webcam. When access is given, a 3-second test audio (or test video) is recorded to ensure that both recording and uploading are working. The recorded media are played back to the participant to ensure that they can be properly heard and/or seen. This procedure can be repeated, if necessary. Upon successful completion of this step, the participant is redirected to the start page of the experimental task, where they are prompted to enter full-screen mode to begin the task.

During the first trial, we usually ask parents to explicitly state their consent for study participation captured using the webcam recording. The consent can be given in a written form, as well. Next, the experimental task is presented. As described above, throughout the task, a small exit button is shown at the lower right corner of the screen, allowing the participant to quit the study at any time. If the study is configured to allow pauses, the participant, upon clicking the exit button, will be redirected to the pause page where they are given the option to resume or terminate the study. The end page informs the participant that they have completed the study and confirms with them that they agree to the processing and use of their data.

### Features

#### Experiment Wizard

**Fig. 2:**
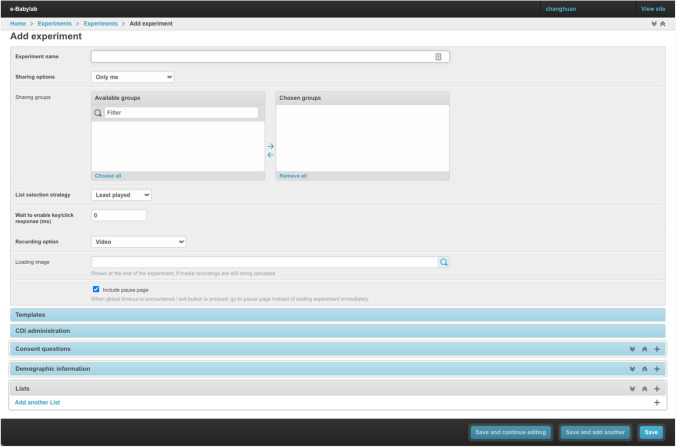
Experiment wizard

At the core of e-Babylab is the Experiment Wizard, a GUI with which an experiment is created (see Fig. 2). The Experiment Wizard consists of six parts: general settings, HTML templates, CDI form, consent form, participant form, and crucially, the experimental task.

##### General settings

In general settings, the basic information related to an experiment (e.g., name, date, and time of creation) is specified. In addition, the access settings, list selection strategy, and recording mode of an experiment are configured here. Specifically, an experiment—including its participants and results—can be made accessible to (a) *owner only* (private), (b) *everyone* (all users), or (c) *group members only* (group-based access control will be detailed later). Thus, administrators of an experiment have complete control over who has access to the experiment. As an experiment can have multiple lists (i.e., versions), three selection strategies allow experimenters to control how the lists (or versions) are distributed across participants: (a) least played, in which the list where the least number of participants have participated is always selected, (b) sequential, in which lists are selected according to the order they are added to an experiment, and (c) random, in which each list has the same probability of being selected, regardless of the number of participants who have participated in a given list. By selecting a recording mode, an experiment can be configured to capture (a) key presses or clicks only, (b) audio and key presses or clicks, or (c) video and key presses or clicks. Note that clicks may represent mouse clicks (when a mouse is used) or touches (when a touchscreen is used). These are recorded as coordinates relative to the browser window, allowing the locations of clicks or touches to be determined. It is also possible to define regions of interest (ROIs) as required (see below, “Experimental task”). The Experiment Wizard also provides the option to include a pause page which may be useful in especially lengthy experiments. In the event that a participant fails to complete an experiment within a given time, or when the exit button is clicked during an experiment, rather than ending the experiment immediately, the participant will be redirected to the pause page, thus giving the participant an opportunity to resume the experiment. Any “pause” events will be recorded in the results.

##### HTML templates

HTML templates allow for the customization of the looks and text (e.g., language) of all experiment webpages, including the welcome page, the consent and participant forms, the microphone and/or webcam setup pages, the experimental task page, the pause page, the error pages, and the end pages. A default set of HTML templates for all experiment webpages are provided for users who do not want to further customize their experiment look (see Appendix 3 for a sample). Alternatively, users can modify the defaults using either the WYSIWYG (What You See Is What You Get) HTML editor or the source code view to provide their own HTML templates as well as Cascading Style Sheets (CSS) files. Customizing these templates also allows for translating the entire experiment to another language.

##### Consent form

This part of the Experiment Wizard allows users to specify consent questions. These will appear on the consent form as mandatory yes/no questions. Since experiments are conducted online and the experimenter may not be physically present to ensure that consent is obtained, e-Babylab automates this by checking that all consent questions are responded to with “yes”. In other words, a participant is only allowed to proceed with an experiment when full consent is obtained. Otherwise, the participant will be redirected to the “Failed to obtain consent” page, which provides an explanation as to why they are unable to proceed with the experiment as well as the option to return to the consent form to change their responses if the responses are provided erroneously or need to be revised.

##### Participant form

In the participant form, personal information can be queried using different types of form fields or questions, including text fields, radio buttons, drop-down lists, checkboxes, number fields, number ranges, age ranges, and sex. Number ranges and age ranges are special field types with automatic checks upon form submission to ensure that the submitted response falls within the specified range. For experiments that include CDI administrations, it is necessary to include “age range” and “sex” fields in the participant form to enable dynamic selection of test items and estimation of participants’ full CDI scores. By setting fields as “required” or “optional,” users can also control which of the form items must be answered before the form can be submitted.

##### CDI form

Short adaptive versions of CDIs, in which items are selected to be maximally informative, using item response theory (Chai et al., 2020), and data from WordBank (http://wordbank.stanford.edu/) as prior knowledge (see Mayor & Mani, 2019), can be administered as part of an experiment, allowing participants’ full CDI scores to be estimated. Real-data simulations using versions of the short form speak to the validity and reliability of these instruments for a number of languages (American English, German, Norwegian, Danish, Beijing Mandarin, and Italian; see Mayor & Mani, 2019, Chai et al., 2020, for further details). Users will need to specify the CDI instrument to be used (detailed later), the assessment type (comprehension or production), and the number of test items to be administered.

##### Experimental task

**Fig. 3:**
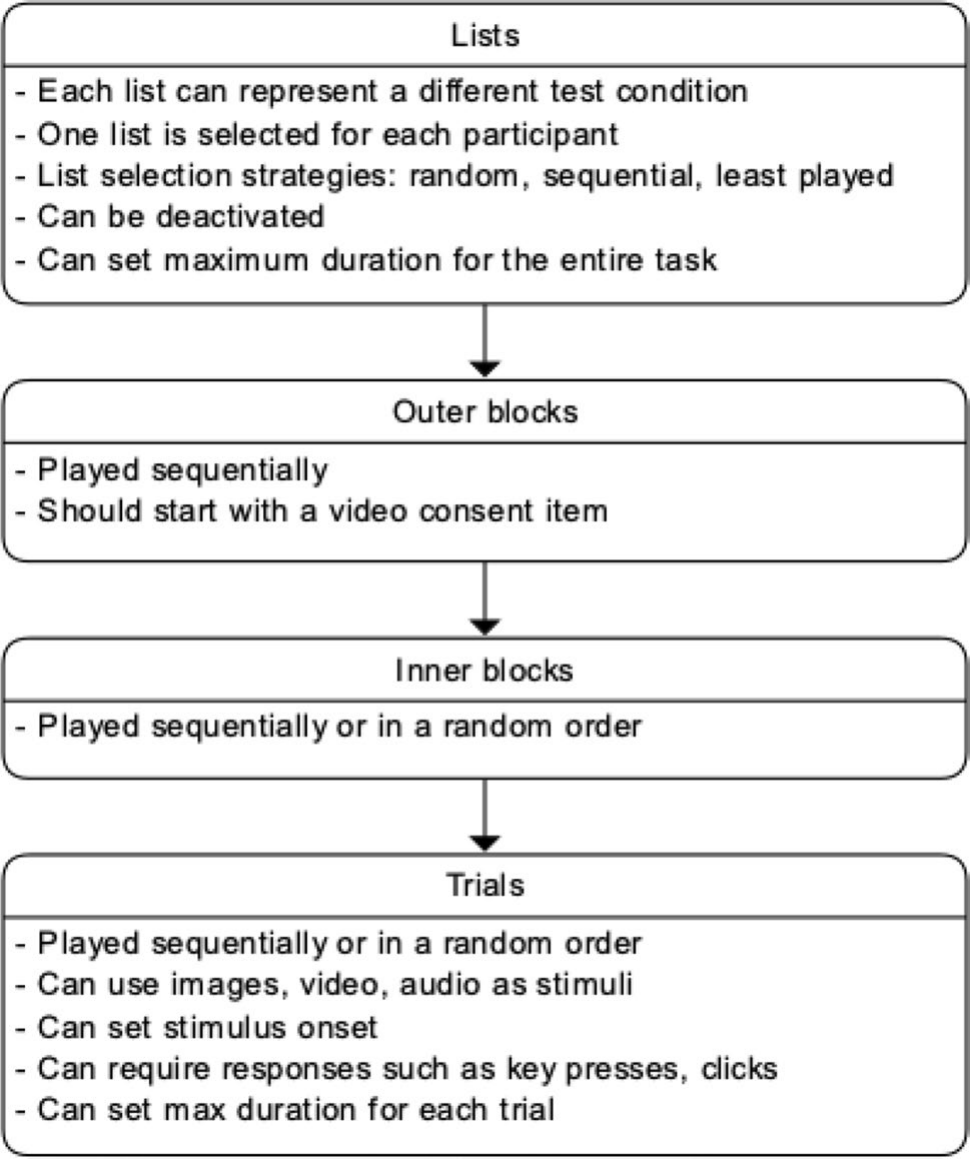
Four-level structure of an experimental task

An experimental task comes with a four-level structure (see Fig. 3). At the first level are *lists*. Each list may represent different versions of the experiment or different conditions of a between-subjects experiment. As each experiment has its own unique URL, an added benefit of having multiple lists instead of multiple experiments is that only a single URL needs to be sent to all participants and the tool automatically distributes participants across the different experimental conditions based on the list selection strategy defined in general settings. Optionally, a list can be temporarily “disabled” to prevent the list from being selected and distributed to future participants; this can be particularly useful when a list has had enough participants and future participants are to be distributed to other lists.

Lists are composed of *outer blocks* which are presented in sequential order, and outer blocks are made up of *inner blocks* which can be presented in either sequential or random order. This increases the flexibility in experimental task design. For instance, when two visual stimuli are to be presented in succession within a single trial, this trial may be represented by an inner block consisting of two trials, each presenting a visual stimulus, in either a fixed or a random order. This flexibility in presentation of stimuli in inner blocks would not be possible without the outer–inner block structure, where we would only be able to present stimuli in either a fixed or a random order, but not both. This is desirable in many experiments where introductory trials (e.g., training, familiarization) typically precede test trials, while test trials, on the other hand, are typically randomized.

At the fourth and most crucial level are *trials* which, as with inner blocks, can be presented either randomly or sequentially. To allow a more granular control over trial setup, the specific responses that are accepted (e.g., clicks, left arrow key, space bar) and the maximum duration of a trial are defined on a trial level. In addition to a visual stimulus (this can be an image or a video), an audio stimulus can be used. Stimuli presentation can be timed by setting the visual and audio onsets in milliseconds (ms). By default, these values are set to 0 so that the stimuli are presented as soon as a trial begins. For experiments involving media recording, users can also decide at a trial level whether media are to be recorded. While knowing the exact click/touch coordinates of a response can be useful, there are times when users are only interested in the general area clicked/touched. By defining a grid layout with *r* rows and *c* columns in each trial, users can establish ROIs on the visual stimulus, so that click/touch responses are recorded as (*r*, *c*). For instance, in a four-alternative forced-choice paradigm, a user would define a 2×2 grid, and a click on the top-left quadrant would be represented as (1,1), the top-right as (1,2), and so on.

### Experiment management

**Fig. 4:**
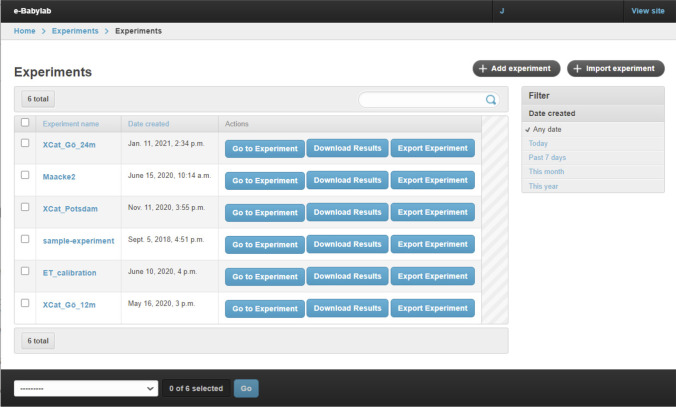
Experiment administration

Experiments are managed through the Experiment Administration interface (see Fig. 4), which presents a list of experiments a user has access to. Through this interface, an experiment setup can be imported and exported. This enables the sharing of experiment setups, which in turn allows experiments to be reused and adapted (e.g., for replications) with minimal effort. The results of an experiment can be downloaded here as well.

### CDI instrument management

**Fig. 5:**
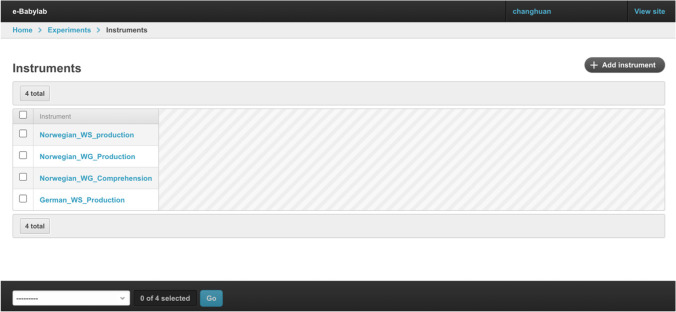
Instrument administration

CDI instruments are managed through the Instrument Administration interface (see Fig. 5), which presents a list of CDI instruments available. An instrument is made up of a set of parameter files required for administering short adaptive versions of CDIs (Chai et al., 2020). To add an instrument, users will need to generate these files using the provided R script that computes the required parameters based on prior CDI data from Wordbank (Frank et al., 2017; see also http://wordbank.stanford.edu/stats for a list of available languages), and subsequently upload these files to e-Babylab.

### Participant management

**Fig. 6:**
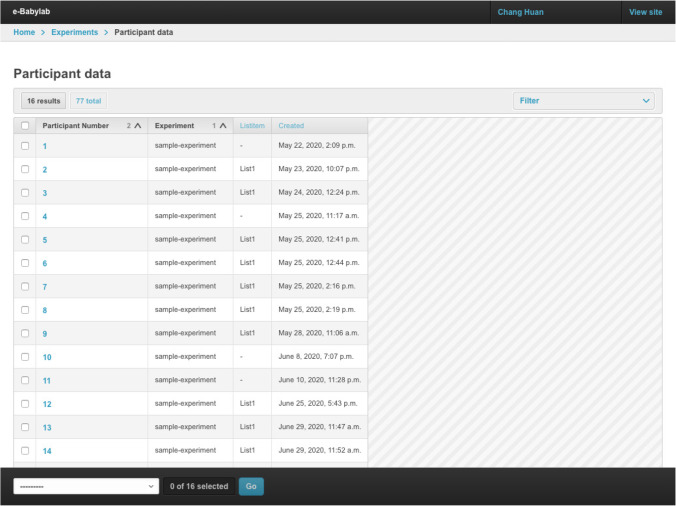
Participant data administration

Participant data are managed through Participant Data Administration, in which a list of participants in all experiments a user has access to is shown (see Fig. 6). By clicking on a participant, users can view the participant’s data, which includes the information provided in the participant form, their screen resolution, participant number, universally unique identifier (UUID; automatically assigned to distinguish participants from different experiments having the same participant number), participation date, and experiment participated in, as well as list assigned. Deleting a participant removes all their data and results.

### Results output

Results are downloaded as a ZIP archive containing an Excel (.xlsx) file for each participant and the media recordings (in .webm format, if any). Each Excel file contains two worksheets. The first contains the participant’s information provided in the participant form, consent form responses, full CDI estimate, CDI form responses, and aspect ratio and resolution of their screen. The second contains information for each trial, including setup information (e.g., stimuli presented, maximum duration allowed), the reaction times, responses given (e.g., keys pressed, mouse click coordinates), screen width and height (for inferring the screen orientation and whether the device has been rotated in each trial), and the file names of associated media recordings.

### File management

**Fig. 7:**
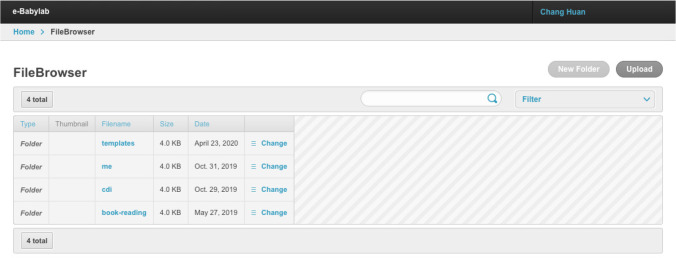
File browser

The tool also features a file browser which allows users to create folders, upload, and manage their own study material, such as audio and visual stimuli, custom HTML templates, and CSS files (see Fig. 7). The supported file types and extensions can be found in Table 1.

**Table 1:** Supported file types and extensions

File type	File extensions
Audio	.mp3, .wav
Document	.css, .csv, .docx, .html, .pdf, .rtf, .tpl, .txt, .xlsx
Image	.gif, .jpeg, .jpg, .png
Video	.mp4, .ogg, .webm

### Authentication and authorization

Access to e-Babylab and its data is secured by authentication and authorization. Authentication verifies the identity of a user and authorization determines the operations an authenticated user can perform on a system (i.e., access rights). Two types of user accounts are offered: normal user and administrator. By default, an administrator has all permissions across e-Babylab (e.g., adding a user, changing an experiment, assigning permissions) without explicitly assigning them. A normal user, on the other hand, does not have any permissions, but instead requires permissions to be assigned by another user who has the permission to do so (e.g., an administrator).

### Security considerations

As a security measure, e-Babylab does not store raw user account passwords. Instead, passwords are hashed using the Password-based Key Derivation Function 2 (PBKDF2) algorithm with a SHA-256 hash (a one-way function recommended in Moriarty et al., (2017)), so that passwords cannot be retrieved. To secure the communication between the e-Babylab server and the client (e.g., browser), e-Babylab is served over Hypertext Transfer Protocol Secure (HTTPS), and any unsecured Hypertext Transfer Protocol (HTTP) requests will be redirected to HTTPS (see “API gateway” in Appendix 2). In addition, media recordings are stored separately from user-uploaded files (i.e., files in e-Babylab’s file browser) such that the only way to access media recordings is by downloading the results of an experiment in Experiment Administration; in other words, users must be logged in and must have access to an experiment in order to access (or download) its results and media recordings.

### Group-based access control

An experiment, including its participant data and results, can be made accessible to other users through groups. For instance, a group can be created for a particular research group or laboratory and an experiment can be shared among all users belonging to this group. As permissions can be assigned on a group level, groups can also be used to more efficiently manage access rights by assigning users to groups. In other words, a user need not be directly assigned permissions, but rather may acquire them through their assigned group(s).

## Selected features of studies successfully run on e-Babylab

An overview of the tasks run on e-Babylab is presented in Table 2. As can be seen in the table, at the time of writing, 12 studies were implemented on e-Babylab, resulting in a total of 1516 children (from 18 months to 6 years) being tested. Below, to illustrate children’s engagement and performance in the tasks, we present a detailed analysis of some data collected via e-Babylab and complement it with some tips that future users may find useful.

**Table 2:** Use of e-Babylab for the studies conducted (2019–Jan. 2022)

Task	Study	Stimuli	Age	*N*	Response type	Trials*	Assessment place	Device	Links to OSF/papers and notes
4AFC	Emotion word recognition	Pictures of faces and audio prompts	2–5 years	200	Touch	8–12	Kindergarten	Samsung Galaxy TabA 10.5″	https://www.psyarxiv/zq6v8
Naming	Emotion word production	Pictures of emotion faces and audio prompts	2–5 years	200	Audio recording	8–12	Kindergarten	Samsung Galaxy Tab A 10.5″	2-year-olds were shy; many refused to name items aloud
4AFC	Word learning via e-book	Pictures of novel objects and audio prompts	2–3 years	55 × 3 times	Touch	12	Kindergarten	Samsung Galaxy Tab A 10.5″	Kartushina et al. (2021)
Naming	Word learning via e-book	Pictures of novel objects and audio prompts	2–3 years	20	Audio recording	8–12	Kindergarten	Samsung Galaxy Tab A 10.5″	Kartushina et al. (2021) 2-year-olds were reluctant to name unfamiliar objects
2AFC	Mutual exclusivity	Pictures of novel and familiar objects + audio	18–20 mos.	25	Touch	20	Lab	Samsung Galaxy Tab A 10.5″	Currently being processed
2AFC	Toddler-based CDI	Pictures of familiar objects + audio	18–20 mos.	25	Touch	48	Lab	Samsung Galaxy Tab A 10.5″	Lo et al. (2021)
2AFC	Online Toddler-based CDI	Pictures of familiar objects + audio	18–36 mos.	138	Touch	48	Asynchronous remote data collection	Tablets, android phones, PC	Lo et al. (2021)
2AFC	Active/passive learning	Pictures of unfamiliar objects	3.5 years	50	Touch	26	Kindergarten	Tablet 1280 × 800	In prep
4AFC	Online word learning	Videos, pictures, and audio prompts	2.5 years	37 × 5 times	Touch	12	At home with parents	Tablets, android phones, PC	Kartushina et al. (2021) Fully online longitudinal (1-week) study
IPL	Prediction task	Pictures of familiar objects + audio	2–8 years	26	Looking behavior	12	Lab	Tablet	Current study
IPL	Categorization task	Videos, pictures, and audio prompts	12 mos.	149	Looking behavior		At home with parents	Tablets, PC	https://osf.io/jc7kv/
IPL	Online word learning	Pictures and audio prompts	2–3 years	139	Looking behavior		At home with parents	Tablets, PC	https://osf.io/8pwqf/

* excluding familiarization trials

### Children’s engagement

To illustrate children’s engagement in the tasks implemented on e-Babylab, we analyzed touch responses in 49 Norwegian 18–20-month-old toddlers performing a two-alternative forced-choice word recognition task, the study referred to as the Toddler-based CDI in Table 2 (Lo et al., 2021). On each trial, two familiar objects (e.g., a dog and a plane) were presented on the screen, followed by a prompt instructing toddlers to select one of them (“Can you touch the dog?”). There were 48 trials in total.

The number of trials in which a touch response was produced, regardless of the accuracy of the response, was used as a measure of toddlers’ motivation to produce a response during the word recognition task. On average, toddlers attempted to provide an answer on 42 out of 48 available trials. The number of touch responses increased with age (*r* = .31, *p* = .03), with 40 and 46 touch responses produced, on an average, by 18-month-old and 20-month-old toddlers, respectively. Trials containing difficult words—those that were known by less than 20% of toddlers as reported by parents in the Norwegian version of the CDI (Simonsen et al., 2014)—elicited fewer touch responses (i.e., 87% of trials *SD = *18) than trials containing easy words (reportedly known by more than 80% of 20-month-old toddlers, i.e., 91% of trials, *SD* = 13), *t*(48) = −2.317, *p* = .0248; Cohen’s *d = *0.249. Anecdotally, around 5% of parents reported having run the task several times, as their child liked to “play” and wanted to do the task again.^4^ These results suggest that toddlers were engaged in the task and that their responses were non-random.

### Home versus lab setting

In the same study (*Toddler-based CDI),* and due to the Covid-19 outbreak, 28 out of 49 toddlers performed the task in their homes, on their parents’ touchscreen devices, as opposed to in the laboratory, thus allowing for a comparison of toddlers’ engagement and accuracy between lab and home/online test settings. A comparison of the number of attempted trials did not provide robust evidence for differences between children who were tested online (*M* = 44, *SD* = 6.3) and in the lab (*M* = 41, *SD* = 7), *t*(40.6) = −1.78; *p* = .083; Cohen’s *d = *0.451. Likewise, a comparison of the number of accurately identified items between the two settings did not provide robust evidence for differences between the two groups of toddlers (online: *M* = 38, *SD* = 7.26 and lab: *M* = 34, *SD* = 8.72), *t*(38.5) = −1.78; *p* = .082; Cohen’s *d = *0.499. In sum, these results suggest there was no robust evidence for differences in accuracy and the degree of motivation to complete the task across toddlers tested in the lab and at home.

### Processing and reaction times

**Fig. 8:**
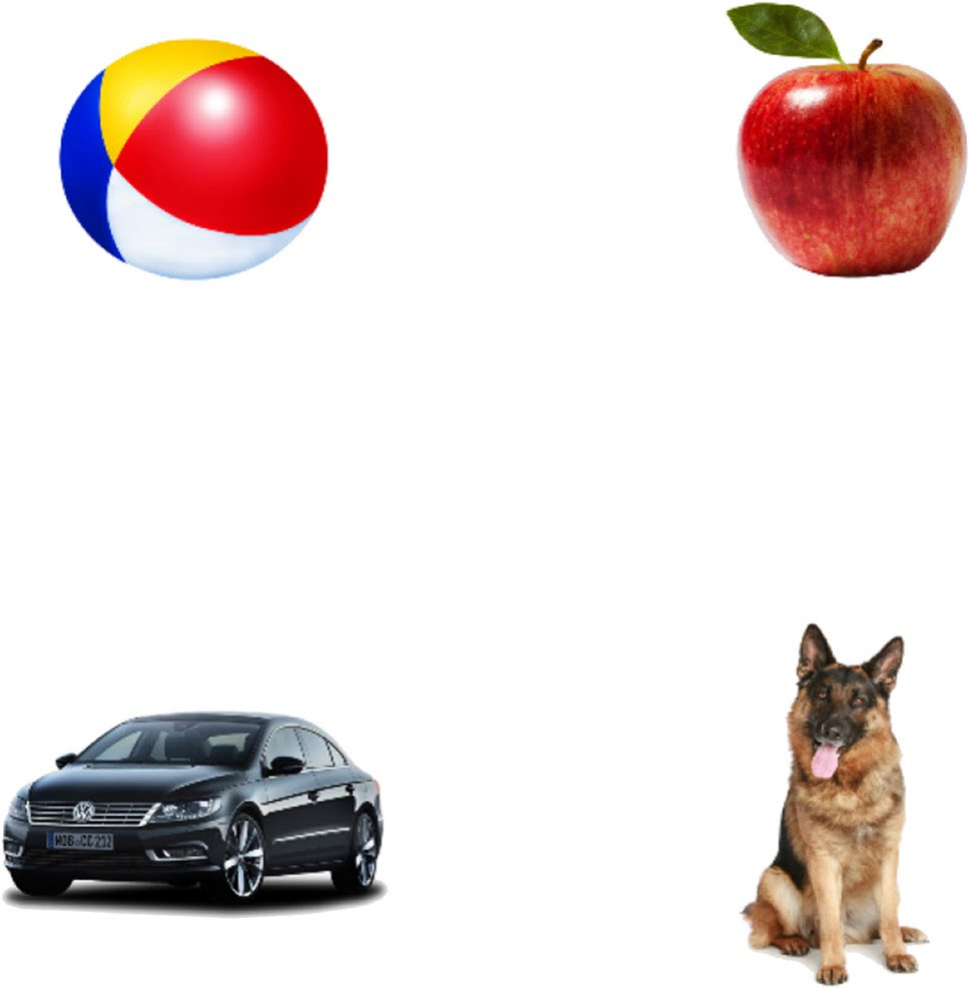
Familiar items used in the four-alternative forced-choice word recognition task

To examine reaction times as a function of age and familiarity with the task, we examined touch latencies for accurate answers in 106 Norwegian 2–6-year-old children (*M* = 3.7 years, *SD* = 1.14 years). These children completed a four-alternative forced-choice word recognition task in their kindergarten. Here, they were presented with four familiar objects (apple, dog, car, and ball, see Fig. 8), used as control trials as part of a larger study. On each trial, children saw four items and were instructed, by an audio prompt, to touch the named target. The timeout was set to 20 s. Children performed the task on a Samsung Galaxy Tab S4, twice in the academic year, in September and June 2019, with a 10-month interval between the two times. Experimenters were instructed not to interfere and were told to never touch the pictures during a trial unless the child’s touch/click was not recorded by the program (i.e., did not launch the next trial). Accidental touches (defined as taking place 1.5 s and less after name onset) were removed from the analyses, in line with previous research (Ackermann et al., 2020). Children’s reaction times ranged from 1.74 to 9.92 s (*M* = 4.15 s, *SD* = 1.64 s).

**Fig. 9:**
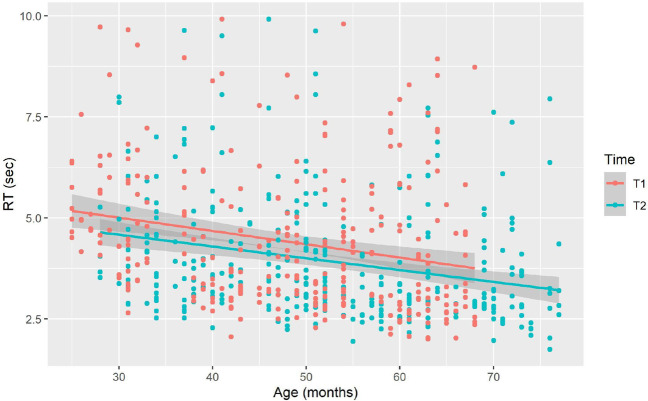
Response latencies at Time 1 and Time 2 across children’s ages

To assess the dynamics of children’s response latencies across time and age, we performed a linear mixed-effect regression model, using *lmer* function in the *lme4* package (Bates et al., 2015), with the fixed factors Time (Time 1 and 2), Age (in months), and Trial; the random factors included Child, adjusted for the effects of Time, and Word.^5^ The dependent variable was log-transformed to meet the assumptions of a normal distribution. The results are summarized in Table 3; the significant effects of Age, Time, and Trial indicate that (1) older children answered faster than younger children, (2) reaction times were faster at Time 2 than at Time 1 (see Fig. 9), and (3) later trials yield faster responses, respectively.

Average reaction times per age and at each testing time are summarized in Table 4. These results indicate that children answered faster at Time 2 than at Time 1, even when age was controlled for. Thus, prior experience with the task and the touchscreen paradigm had long-lasting beneficial effects on reaction times when performing the task for the second time. The effect of prior experience with the task varied across ages and ranged between 0.18 and 0.68 s. A very recent study has similarly reported that experience with paradigms affects infants’ behavior in the task (e.g., more experience with the head-turn preference paradigm; more lab visits), leading to smaller familiarity preference (i.e., smaller effect sizes), suggesting that prior experience with the task accumulates and modulates the learning outcome (Santolin et al., 2021).

**Table 3:** LMM results for (log-transformed) reaction times

	*β*	*SE*	*DF*	*t*	*p*
Intercept	1.955	0.075	156.439	25.794	< 0.001 ***
Age	−0.008	0.001	101.358	−6.149	< 0.001 ***
Time 2	−0.083	0.035	102.043	−2.366	0.0198 *
Trial	−0.055	0.010	469.126	−5.047	< 0.001 ***

**Table 4:** Descriptive statistics for response latencies as a function of time and age

Age (years)	Time	*N*	Mean (s)	95% CI
2	T1	16	5.26	[4.83, 5.69]
2	T2	11	4.58	[3.95, 5.20]
3	T1	21	4.53	[3.86, 5.21]
3	T2	20	4.35	[3.96, 4.75]
4	T1	21	4.28	[3.81, 4.76]
4	T2	22	3.76	[3.34, 4.17]
5	T1	18	4.07	[3.42, 4.72]
5	T2	20	3.54	[3.15, 3.92]
6	T2	8	3.53	[2.64, 4.43]

#### Codability of looking time data

A number of paradigms with young infants rely on collecting data of infants’ eye movements as an implicit measure of children’s processing of audio and visual information presented in the study. A seismic change in this regard was brought about with the introduction of the intermodal preferential looking paradigm, where children were presented with audiovisual input and their eye movements across a screen were analyzed as an index of their recognition of their relationship between the auditory and visual input (Golinkoff et al., 1987). e-Babylab allows for this by requesting participants’ permission to access their webcam and/or microphone and capturing video or audio of participants on a trial-by-trial basis during the study.

We capitalized on this possibility for an online replication of a standard looking time task examining whether young children can use thematic information provided in the input to anticipate upcoming linguistic input and use this to fixate a target image prior to it being explicitly named (Mani & Huettig, 2012).^6^ Thus, here, participants are presented with images of two familiar name-known objects (e.g., a cake and a bird) and hear the sentence “The boy eats the big cake” or “The boy sees the big cake.” The verb “eat” thematically constrains how the sentence will be continued, with only edible nouns constituting permissible continuations of the input thus far. If children are sensitive to such thematic constraints and can use them to anticipate upcoming linguistic input, we expect them to fixate the target object “cake” soon after the verb “eat” is presented, but not when the verb “see” is presented.

We included here data from 25 participants aged 2–8 years (*M* = 55 months, *SD* = 16 months, range: 28–101 months). Participants were tested at a laboratory testing facility, as the focus of this study was to examine the usability of the looking time data collected. The stimuli and setup of the task were identical to that used in Mani & Huettig (2012). The videos for each individual trial for each participant were coded by two coders who were blind to the trial conditions. Videos were coded in ELAN^7^ (Lausberg & Sloetjes, 2009). Since we were interested in the usability of video data obtained and the extent to which we could reliably code where participants were looking at any point in the trial, the two blind coders coded all trials in the study as to whether children were looking to the left or right side of the screen or away from the screen. Cohen’s kappa for inter-rater reliability suggested almost perfect agreement between raters, *κ = *.993, *p* < .001, indicating that the quality of video data collected was adequate to allow reliable coding of whether participants were looking to the left or right of the screen.

We coded video data for 297 of 312 trials presented to children (26 participants each presented with 12 trials), with a mean of 11.42 trials per participant (*SD* =1.73, range: 4–12). The video data of two participants were incomplete (4 and 8 trials missing, respectively) because of technical errors during the video transfer. Three additional trials were not included in the analysis due to coder error or technical error during coding. And the data of one child (12 trials) were excluded due to technical error (see above), resulting in 285 trials for analysis. During the critical time windows, participants spent 89% of the time looking at either the target (56%) or the distractor (35%) and only 8% of the time not looking to either the left or right of the screen, suggesting that they were paying attention to the information being presented. When considering the data across the entire trial, they spent 27% of the time looking at the distractor, 41% looking at the target, and 33% looking at neither the target nor distractor.

**Fig. 10:**
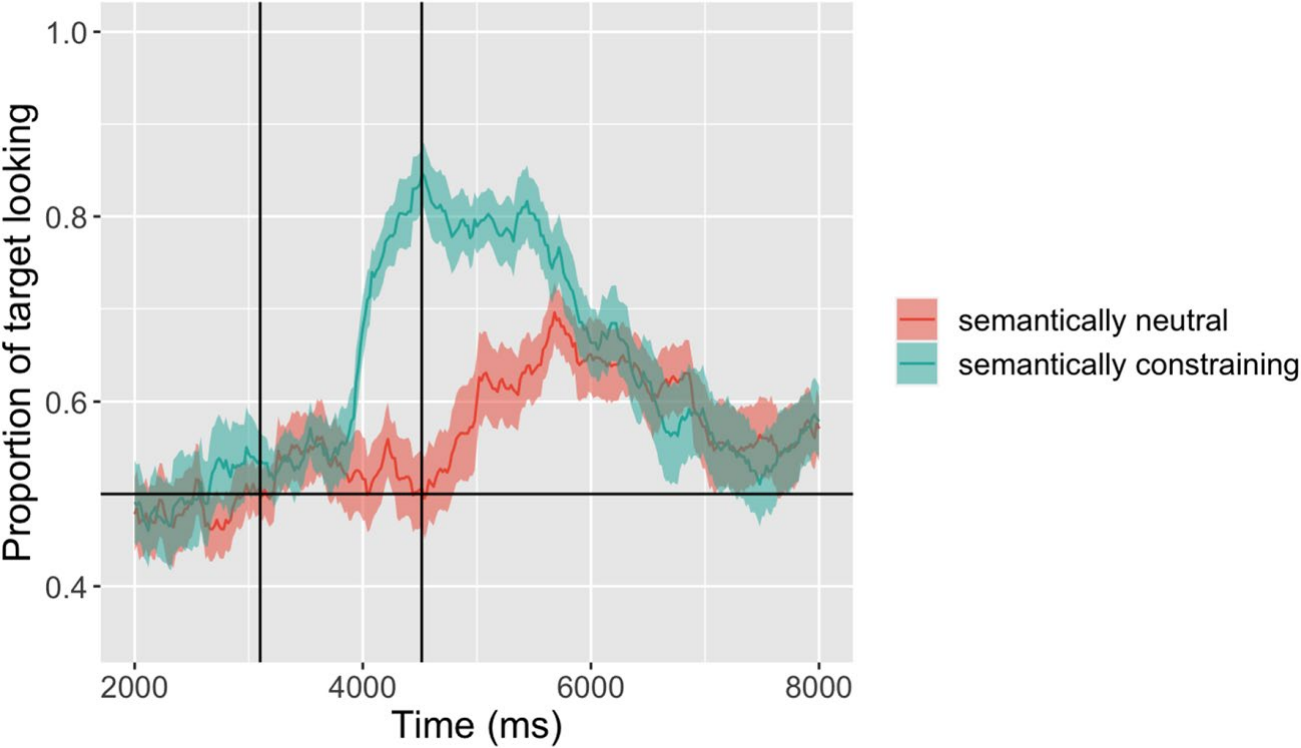
Time course of fixations to the target across the critical time window in the two conditions. Vertical lines indicate the onset of the verb (at 3000 ms) and the earliest onset of the noun (> 4000 ms)

We replicated the findings of the original study (Mani & Huettig, 2012, see Supplementary Information for further details). Figure 10 shows the time course of fixations to the target across the critical time window. As Fig. 10 suggests, children fixated the target object *cake* soon after they heard the verb “eat” but not after hearing the verb “see.” This replication of the results of the original study highlights the viability of e-Babylab for such fine-grained studies examining the dynamics of infants’ eye movements across the screen over time. The timing of the effect in Fig. 10, a few hundred milliseconds after the onset of the verb, in particular highlights the efficacy of the tool even for studies examining rapidly changing stimuli like speech processing.

#### Looking time studies at home

One of the caveats of the study mentioned above is that participants were tested online in a laboratory with a stable internet connection. It is, therefore, important to know to what extent similar data quality is to be expected when children are tested in the comfort of their own homes. With this in mind, we highlight two additional online looking time studies that we have run using the same tool. In one study, we presented infants with multiple objects from two categories and then examined infants’ categorization of the objects to the extent that they distinguished between a novel object of one of the familiarized categories and an unfamiliar object from a different category (Bothe et al., in prep). Each child was presented with ten 10-second-long training trials and two 10-second-long test trials (and three attention-getters that we do not report on further). In one study, 149 one-year-olds were presented with 1730 trials altogether, from which we obtained video data for 1368 trials (70.8%) and were able to reliably code data for 1348 trials (98.54% of the trials for which we obtained video data). Inability to code was typically due to poor lighting or the child not being positioned properly (e.g., not showing both eyes clearly). In an initial test (57.8% of the dataset), video stimuli presented during the test were too large (1.5 MB–7.2 MB), leading to considerable data loss (i.e., we only obtained 57% of video data from a total of 1000 presented trials). We resolved these initial data loss issues by reducing the size of the video stimuli. Following compression of video stimuli (354.2 KB–1.3 MB), we obtained 91.08% of video data (from 639 trials) for the remaining 730 trials. The size of video files in the initial test accounted for 82.08% of data loss reported above, which was reduced to only 17.91% data loss once the video files were compressed. In another study, we presented 52 children with 22 trials (that needed to be coded) and an additional 12 trials for which we did not need to code the data (34 trials altogether). We obtained video data for 1584 out of 1768 trials (89%). We excluded 12 children from further coding (23% dropout) because they did not provide us data for critical trials in the study, leaving us with 40 children who provided us with 880 trials that needed to be coded. Of these, we could reliably code 851 trials (96%). Altogether, this suggests a relatively high dropout rate (more on this below) of around 25% of children whom, across the looking time studies, we were unable to include in further analyses. Nevertheless, notwithstanding high rates of video loss and dropouts, the data obtained from e-Babylab can be reliably coded, as between 82% and 96% of trials were included in the analyses,^8^ suggesting acceptable efficacy of e-Babylab in children’s natural environments.

## Tips for improving data quality in e-Babylab

Having now run 1516 children in studies on e-Babylab, we have collected a number of suggestions that improve data quality in studies using the platform. We now routinely implement these measures in our study protocols for e-Babylab. Here we briefly list these for future users of e-Babylab.

1. For designs containing audio stimuli, it is important to let participants adjust the volume before the test. For this purpose, we included a trial with an audio playing continuously and with no timeout at the beginning of each task, and instructed participants to adjust the volume to a level suitable for themselves and their child.
2. For tasks performed remotely and involving the use of handheld devices (e.g., tablets, smartphones), participants (or caregivers) need to be instructed at the beginning of the study to hold their device in either portrait or landscape display mode (depending on the design of the task) and to not re-orient the device during the task.
3. Participants should be informed before they are directed to the experiment URL that they need to open the experiment with a compatible browser (i.e., Chrome or Mozilla Firefox) and device (e.g., no iPads—see “Media recording” in Appendix 2).
4. We recommend the use of Handbrake (https://handbrake.fr/) for optimizing video stimuli for web pages and converting these to formats that keep the file sizes small without compromising on quality (e.g., .mp4). A stable internet connection is a prerequisite for participating in an e-Babylab study. Internet data usage varies depending on the size of the study. For instance, a study that uses video stimuli and records videos of participants will require much more data than one that presents images and records only screen touches.
5. Some of our projects involved the recording of participants’ eye movements. We noted that we suffered some data loss due to coders not being able to see the child’s eyes or assess where the child was looking. Here, we suggest it might be good to inform the parent beforehand that it is important that we are able to clearly see the child’s eyes and to ask them to ascertain this during the video check at the beginning of the study. Instructions sent to parents could also include specific details with regard to lighting in the room where the study takes place.
6. Sometimes, we also experienced issues with video recordings not being uploaded to the server. We found this issue to occur more frequently when single trials were rather long. Therefore, we recommend keeping trials short (ideally less than 30 s) or, if possible, splitting longer trials into a sequence of several shorter trials within an outer block

### Planned features

A series of features are being evaluated or planned to make e-Babylab even more user-friendly and efficient. At the time of writing, they are not part of the release: (1) integrate and adapt WebGazer (Papoutsaki et al., 2016) to allow (a) automatic real-time gaze detection using participants’ webcam, so that gaze data can be directly obtained, thus obviating the need for manual gaze coding and for transferring video data to the server, and (b) self-calibration based on participants’ gaze (instead of clicks) to better suit its use with children and infants, (2) reduce the necessary bandwidth when participants have reduced internet speed by potentially delaying participant video upload until the end of the study, thereby reducing data loss and potential lags between video stimulus presentation and video recording, (3) allow greater degrees of freedom with regard to counterbalancing and randomization of trials, (4) allow for different kinds of responses (e.g., video/audio recordings) to be collected in different trials, and finally, (5) integrate adaptive structures (e.g., if participant responds with Y, go to trial n). We are currently working on implementation of these changes and beta-testing once these changes have been implemented so that we can hopefully present a one-stop tool to developmental researchers.

## Conclusion

Here, we present a highly flexible tool that allows researchers to create and conduct online studies using a wide range of measures. We also demonstrate the efficacy of the tool with regard to the data quality. Importantly, we highlight a number of use cases for e-Babylab particularly in developmental research. With regard to the aforementioned criteria for an interface suitable for developmental research, e-Babylab allows the possibility of recording webcam videos of the test sessions and brings looking time tasks to the child’s home. In addition, e-Babylab is browser-based and thus does not require participants to install additional software or to possess extensive computer know-how to be able to take part in studies. Further, the data collected are stored on local university servers (of the respective groups using e-Babylab). We believe this latter point is particularly important in developmental research, since parents of young children may have reservations concerning data security. Finally, given that many researchers do not have the required skills to independently program online studies, e-Babylab provides a highly intuitive graphical user interface that allows even those without programming skills to conduct online studies. e-Babylab, therefore, provides a solution to bringing developmental research online and offers opportunities to reach a wider population in developmental studies, at least in settings where access to high-speed internet and devices is ensured.

## Figures and Tables

**Fig. 11:**
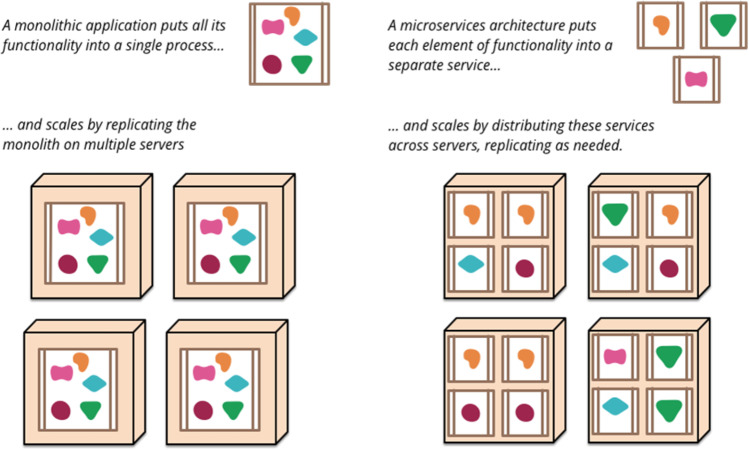
Monoliths and microservices (taken from Lewis & Fowler, 2014)

**Fig. 12:**
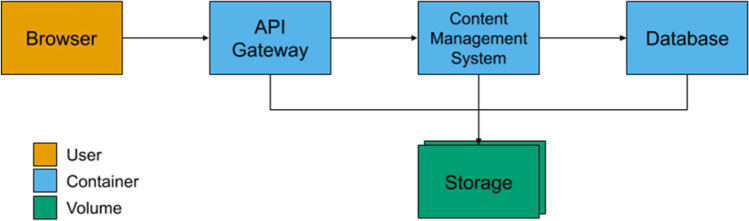
Components of e-Babylab

**Fig. 13:**
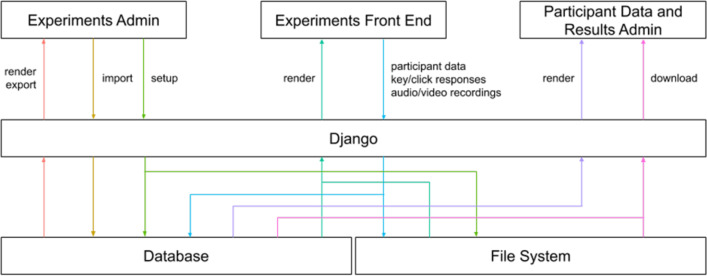
Data flow in the content management system

## Appendix 1. Overview of actively maintained tools for unmoderated online testing

**Table 5:** X indicates function not possible or not required, √ indicates function possible and/or required, while – indicates not applicable

Tool	Webcam recording possible	Browser-based	Data storage location	Programming required		Main functions		Key features	Open source	Cost
				Common tasks	Complex tasks	Build studies	Host and run studies			
Discoveries Online Rhodes et al. (2020)	√	√	USA	-	-	X	√	Video recording, guide on steps to take in-person studies online	X	-
Gorilla Anwyl-Irvine et al. (2020)	Under beta-testing	√	EU	X	X	√	√	Three different GUIs for building questionnaires, tasks, and study logic, code editors for further customization, integration with participant recruitment platforms, ready-to-use templates for common tasks, audio recording, mouse tracking	X	Paid
Inquisit Web https://www.millisecond.com/products/inquisit6/weboverview.aspx	X	X	EU / USA	√	√	√	√	Dedicated scripting language, ready-to-use templates for common tasks, high timing accuracy and precision with the Inquisit Player app (downloadable)	X	Paid
JATOS Lange et al. (2015)	X	√	Local server	-	-	X	√	GUI (instead of CLI) for communicating with the server and the database, straightforward to setup locally, compatibility with study builders (e.g., OpenSesame / OSWeb, jsPsych, lab.js)	√	Free
jsPsych de Leeuw (2015)	√	√	-	√	√	√	X	JS library providing pre-programmed components needed to build studies (e.g., measure RT, display stimuli, mouse tracking), integration with participant recruitment platforms	√	Free
LabVanced Finger et al. (2017)	√	√	EU	X	X	√	√	GUI for building, hosting, and managing studies, support for real-time multiplayer tasks, audio and video recording, ready-to-use templates for common tasks, integration with participant recruitment platforms	√	Paid*
lab.js Henninger et al. (2021)	X	√	-	X	√	√	X	GUI for building studies, code editor for further customization, custom logic, and complex tasks	√	Free
Lookit Scott & Schulz (2017)	√	√	USA	√	√	√	√	Video recording, templates for common tasks, JS or JSON for custom tasks	√	-
MindProbe (based on JATOS) https://mindprobe.eu/	X	√	EU	-	-	X	√	(See JATOS above)	√	Free
Open Lab https://open-lab.online/	X	√	EU	-	-	X	√	GUI for hosting, running, and managing studies created using lab.js	√	Paid*
OpenSesame / OSWeb Mathôt et al. (2012)	X	√	-	X	√	√	X	Desktop application provides GUI for building online studies (JS) and offline studies (Python), code editor for further customization, custom logic, and complex tasks	√	Free
Pavlovia	X	√	UK	-	-	X	√	GUI for hosting, running, and managing studies created using PsychoPy / PsychoJS, jsPsych, and lab.js, repository for sharing online studies and the code base	X	Paid
PsychoPy / PsychoJS Peirce et al. (2019)	√	√	-	X	√	√	X	Desktop application provides GUI for building offline studies (in Python) which can be exported as online studies (in JS), code editors for further customization, custom logic, and complex tasks	√	Free
PsyToolkit Stoet (2017)	√	√	EU / USA	X	√	√	√	Ready-to-use templates for common tasks, dedicated scripting language (simpler than JS) for further customization, custom logic, and complex tasks	√	Free
Tatool Web von Bastian et al. (2013)	X	√	UK	X	√	√	√	GUI for building studies, ready-to-use templates for common tasks, code editor for further customization, custom logic, and complex tasks	√	Free
Testable Rezlescu et al. (2020)	X	√	EU	X	X	√	√	Build studies by filling in a natural language form, further customization, custom logic, and complex tasks by filling in a spreadsheet, ready-to-use templates for common tasks, Testable Minds (Testable’s own participant pool), support for real-time multiplayer tasks	X	Paid

^*^ Basic plan which includes one study is available for free. EU: European Union, UK: United Kingdom, USA: United States, JS: JavaScript, GUI: graphical user interface, CLI: command line interface, RT: reaction time

## Appendix 2. Technical underpinnings of e-Babylab

### Microservices and docker

e-Babylab is developed using a microservices architecture. Contrary to the commonly used monolithic architecture where all the components of an application are developed as a single entity and run in the same process, the microservices architecture centers on developing an application as a set of lightweight and loosely coupled services (or small applications), each running in its own process and serving a specific purpose (see Fig. 11; Lewis & Fowler, 2014). As a result, services of the same application can be developed, deployed, and maintained independently—and rapidly. The independence of services also means that the failure of a single service will not affect other services (i.e., the rest of the application remains functional). Moreover, services can be reused and applied to other applications, thus reducing development costs.

Microservices lend themselves well to operating system-level virtualization (also known as containerization), which involves bundling the application code with all its libraries, system tools, configuration files, and dependencies (with the exception of the operating system) so that the application will always run the same, regardless of the computing environment (IBM Cloud Education, 2019). Such bundles, referred to as containers, are lightweight in that they share the host machine’s operating system kernel, effectively eliminating the overhead of running multiple operating systems. This further translates into faster start-up times and smaller memory footprints. For these reasons, Docker (https://www.docker.com), an open-source, lightweight container virtualization platform that runs on Mac, Windows, and Linux, is chosen to deploy the e-Babylab services.

As shown in Fig. 12, e-Babylab is built of three services—the application programming interface (API) gateway, the content management system, and the database—each encapsulated in a container. The arrows represent dependencies between services, which are started in dependency order. In other words, the database is started before the content management system, and lastly, the API gateway. As containers are ephemeral, such that they can be stopped, destroyed, rebuilt, and replaced as needed, data generated or used by containers do not persist when the containers are destroyed. Thus, data that need to be persisted are stored in volumes managed by Docker on the host machine. In this way, containers can easily be replaced (e.g., in upgrading a service) without any loss of data.

Apart from containers, the Docker architecture includes two other major components, namely images and registries. Briefly, containers are created from images which serve as blueprints. Each image is defined by a Dockerfile that contains the instructions to create a given image. During a build process, the instructions in a Dockerfile are executed and stored as an image. For ease of distribution and sharing, images can be pushed to registries where images are stored. The Docker-Compose file specifies whether images are to be pulled (i.e., downloaded) from a registry or built locally (using a Dockerfile). The API gateway and the database images of e-Babylab are pulled from Docker Hub (i.e., Docker’s public registry), as they can be used as is. On the other hand, as the content management system is heavily customized, the image is built locally.

To orchestrate these services (i.e., to automatically configure, coordinate, and manage them) and start up e-Babylab, Docker Compose is used. By running *docker-compose up*, Docker Compose pulls the images for the API gateway and the database, builds an image for the content management system, and finally starts and runs the e-Babylab services as defined in the Docker-Compose file.

### API gateway

The API gateway is implemented using the open-source version of NGINX (https://hub.docker.com/_/nginx), a multipurpose web server which also acts as a reverse proxy and Transport Layer Security (TLS) terminator. The API gateway acts as the entry point into e-Babylab and forwards a client’s (e.g., browser) requests to the content management system and database services. With the addition of a TLS certificate, this entry point is protected by TLS, the successor to Secure Sockets Layer (SSL), which takes care of securing end-to-end communications (e.g., data transfer) between two systems. Put simply, e-Babylab is served over Hypertext Transfer Protocol Secure (HTTPS). Additionally, NGINX is configured to redirect any unsecured Hypertext Transfer Protocol (HTTP) requests to HTTPS.

### Content management system

#### Django

The content management system which provides the administrative interface to manage experiments as well as participant data and results is implemented with Django (https://hub.docker.com/_/django), an open-source Python-based web framework. With its aim to encourage rapid development, Django provides a complete set of ready-made components needed in most web development tasks, such as the authentication system and the dynamic administrative interface described earlier. On top of the aforementioned TLS/HTTPS protection, Django provides an extra layer of security by preventing most common security vulnerabilities in web applications, such as cross-site scripting, cross-site request forgery, Structured Query Language (SQL) injection, and clickjacking (see The OWASP Foundation, 2017, for more details on common security vulnerabilities). Thus, focus can be placed on developing the parts of a project that are unique, which in the case of e-Babylab are the experiments as well as participant data and results.

In order to generate HTML dynamically, for both e-Babylab and the experiment front end, Django’s own template system, namely the Django template language, is used. Typically, a template contains both static (non-editable) and dynamic (editable) parts of the desired HTML output, allowing the same design to be reused while the content changes dynamically. As shown in Fig. 13, Django retrieves data from the database and the file system—where template files, stimuli, and media recordings are stored—and renders (i.e., interpolates) the templates with these data to dynamically display contents on the user-facing administration system and the participant-facing experiment front end. The figure also shows the flow of data in setting up, importing, and exporting experiments; in recording participant data and responses during an experiment; and in downloading participant data and results.

### Import and export of an experiment setup

The import and export functions are realized using JavaScript Object Notation (JSON), a lightweight, human-readable, text-only data interchange format used in storing and transporting data. For exporting an experiment setup, all parts of the experiment setup, from the general settings until the trials, are first retrieved from the database and serialized into JSON objects, which are then downloaded as a single JSON file. Likewise, for importing an experiment setup, a user-uploaded JSON file containing JSON objects making up the parts of an experiment setup is simply deserialized and a new experiment is created, set up, and stored in the database.

### Media recording

An important feature offered in experiments created with e-Babylab is the capability of recording audio and video. This is enabled by the MediaStream Recording API (https://developer.mozilla.org/en-US/docs/Web/API/MediaStream_Recording_API). As the API is only available in Google Chrome and Mozilla Firefox for Android and desktop, experiments programmed with e-Babylab will not run on current iOS devices, such as iPhones and iPads. For this reason, a browser compatibility check is included as part of every experiment.

Media recording involves both the front end and the back end. On the front end, the getUserMedia() function of the MediaDevices interface asks for permission to use the participant’s media input devices (e.g., microphone and/or webcam) and produces a MediaStream object containing audio or video tracks, depending on the type that is requested. This MediaStream object is then passed to a MediaRecorder object which is configured to record media as 1-second chunks to be uploaded via Asynchronous JavaScript and XML (AJAX) to the Django back end. Media are recorded per trial. When the final chunk of a trial is received on the back end, individual chunks are merged as a single media file which is then stored on the file system and referenced in the database. The approach of uploading media recordings as 1-second chunks obviates the need for restarting the upload for the entire media file, in case of a network interruption or some other transmission failure. To further account for low-bandwidth environments, videos are recorded in 640 × 480 pixels.

### Database

The database where experiments as well as participant data and results are stored is a relational database created using the open-source relational database management system PostgreSQL (https://hub.docker.com/_/postgres). In a relational database, data are stored in tabular form, where rows are referred to as *records* and columns as *attributes*. Records in different tables can be linked—or related—based on a unique *key* attribute. With this key, data from multiple tables can be retrieved with a single query. For instance, downloading participant data and results of an experiment requires data to be retrieved from the participant data table, the experiments table, the lists table, the outer-blocks table, and so on. This can be easily achieved using the experiment identifier (ID) which serves as the key. In addition, as PostgreSQL is supported by Django, any changes made to the database schema, such as the addition of new tables, can simply be stored by running *python manage.py makemigrations* which automatically generates the SQL commands needed to modify the database schema. To execute these commands (i.e., to apply the changes) the *python manage.py migrate* command is used (see “Django documentation: Migrations, 2019 for more details on Django migrations).

## Footnotes

1 https://manybabies.github.io/MB-AtHome/

2 Desktop here refers to desktop and laptop computers running on Microsoft Windows, macOS, or Linux.

3 To prevent bots from taking part in studies, reCAPTCHA v3 verification (https://developers.google.com/search/blog/2018/10/introducing-recaptcha-v3-new-way-to), in which no user interaction is required, is run upon participant form submission.

4 Note that only the first attempt was used for the analyses.

5 The model including a Time/Stimulus random slope did not converge.

6 This study was run on a previous version of e-Babylab.

7 ELAN (Version 5.9) [Computer software]. (2020). Nijmegen: Max Planck Institute for Psycholinguistics, The Language Archive. Retrieved from https://archive.mpi.nl/tla/elan

8 We do not provide details of the results of these studies due to the data being privileged to written manuscripts of these studies.
